# Radiomic Features as Artificial Intelligence Prognostic Models in Glioblastoma: A Systematic Review and Meta-Analysis

**DOI:** 10.3390/diagnostics14212354

**Published:** 2024-10-22

**Authors:** Dewa Putu Wisnu Wardhana, Sri Maliawan, Tjokorda Gde Bagus Mahadewa, Rohadi Muhammad Rosyidi, Sinta Wiranata

**Affiliations:** 1 Neurosurgery Division, Department of Surgery, Faculty of Medicine, Universitas Udayana, Udayana University Hospital, Denpasar 80361, Indonesia; 2 Neurosurgery Division, Department of Surgery, Faculty of Medicine, Universitas Udayana, Prof. Dr. IGNG Ngoerah General Hospital, Denpasar 80113, Indonesia; 3 Department of Neurosurgery, Medical Faculty of Mataram University, West Nusa Tenggara General Hospital, Mataram 84371, Indonesia; 4 Faculty of Medicine, Universitas Udayana, Denpasar 80232, Indonesia

**Keywords:** glioblastoma, radiomic features, artificial intelligence, overall survival, progression-free survival

## Abstract

Background: Glioblastoma, the predominant primary tumor among all central nervous systems, accounts for around 80% of cases. Prognosis in neuro-oncology involves assessing the disease’s progression in different individuals, considering the time between the initial pathological diagnosis and the time until the disease worsens. A noninvasive therapeutic approach called radiomic features (RFs), which involves the application of artificial intelligence in MRI, has been developed to address this issue. This study aims to systematically gather evidence and evaluate the prognosis significance of radiomics in glioblastoma using RFs. Methods: We conducted an extensive search across the PubMed, ScienceDirect, EMBASE, Web of Science, and Cochrane databases to identify relevant original studies examining the use of RFs to evaluate the prognosis of patients with glioblastoma. This thorough search was completed on 25 July 2024. Our search terms included glioblastoma, MRI, magnetic resonance imaging, radiomics, and survival or prognosis. We included only English-language studies involving human subjects, excluding case reports, case series, and review studies. The studies were classified into two quality categories: those rated 4–6 were considered moderate-, whereas those rated 7–9 were high-quality using the Newcastle–Ottawa Scale (NOS). Hazard ratios (HRs) and their 95% confidence intervals (CIs) for OS and PFS were combined using random effects models. Results: In total, 253 studies were found in the initial search across the five databases. After screening the articles, 40 were excluded due to not meeting the eligibility criteria, and we included only 14 studies. All twelve OS and eight PFS trials were considered, involving 1.639 and 747 patients, respectively. The random effects model was used to calculate the pooled HRs for OS and PFS. The HR for OS was 3.59 (95% confidence interval [CI], 1.80–7.17), while the HR for PFS was 4.20 (95% CI, 1.02–17.32). Conclusions: An RF-AI-based approach offers prognostic significance for OS and PFS in patients with glioblastoma.

## 1. Introduction

Glioblastoma is the predominant primary tumor among all central nervous system cancers, accounting for around 80% of cases [1]. It continues to be an untreatable condition, with a median lifespan of about 15 months [2]. Just 5.5% of patients manage to survive for 5 years after being diagnosed [3]. The global prevalence data show that the annual incidence rate ranges from 0.59 to 5 per 100,000 individuals. Data are collected from multiple nations, including the United States, Australia, Britain, Korea, Greece, and Jordan, based on events specific to the age per 100,000 individuals (using the ICD-O 9450 morphological code) [4,5,6,7,8].

Prognosis in neuro-oncology entails assessing the advancement of the disease in different persons, considering factors such as the disease stage, its location, and the intended treatment or surgery. The crucial criteria to consider as a reference are overall survival (OS) and progression-free survival (PFS), guiding factors in determining subsequent treatment. Due to the difficulties in early detection and the invasive characteristics of tumor cells, completely removing them by surgery is a considerable problem [9].

The current conventional therapy involves the excision of the tumor through surgery, followed by the application of radiation and chemotherapy. Despite advancements in surgical imaging techniques that allow for more thorough tumor tissue removal, it is essential to balance aggressive tumor resection with the preservation of brain function and the overall well-being and quality of life of patients. Notably, patients with glioblastoma from lower socioeconomic backgrounds are less frequently tested for O6-Methylguanine-DNA-methyltransferase (MGMT) [10]. Failure to perform MGMT exams can lead to consequences of distorted prognoses and result in diagnoses at more advanced stages, with larger and more complex tumors. Furthermore, this particular group of people is rarely given a variety of treatment methods, resulting in decreased chances of survival. Nevertheless, these measures in disease treatment are fraught with difficulties, and mistakes can result in patient illness and death [11]. The problems encompass the requirement for accurate disease diagnosis and staging to inform clinical decisions, the ongoing monitoring of disease progression after therapy, which might be hindered by signals from nearby brain tissue, and the increasing importance of finding genetic patterns [12]. The genetic patterns have a significant influence on tumor behavior and clinical consequences [13]. The difficulties in managing glioblastoma stem from several factors, such as the intricate nature of the brain, restricted availability of precise imaging and biopsy techniques, the inherent diversity in tumor biology (genetic heterogeneity, epigenetic modifications, the tumor microenvironment, tumor evolution, and interactions with the immune system), varying rates of progression, individual differences in treatment response, and the lack of dependable biomarkers for predicting prognosis [14,15]. The neurological tissue’s susceptibility to conventional therapeutic methods, such as surgery, radiation, and chemotherapy, adds to the complexity of their management [16].

Artificial intelligence (AI) is a valuable tool for medical professionals in choosing treatment procedures. AI showcases its potential in brain tumor management through its ability to expedite and improve MRI imaging, identify abnormalities, optimize workflows, provide precise measurements, analyze vast amounts of medical imaging data, and identify patterns that may not be readily noticeable to human observers [17,18]. It has greatly enhanced the area by offering comprehensive image analysis for diagnostics, tumor classification, prognosis prediction, and the assessment of treatment response [19,20]. Additionally, it aids in planning surgical and nonsurgical treatments, expedites the discovery of new drugs, and assists in monitoring the recurrence of medical conditions. AI tools can be integrated into clinical trials to enhance patient outcomes and potentially lead to tailored therapy [13,21]. AI is essential in clinical neuroimaging for tasks including accurately identifying tumor boundaries and kinds, improving pre-therapeutic planning, and evaluating post-therapeutic responses [22]. The ability of AI to analyze large datasets presents a revolutionary method for precision medicine, potentially addressing common challenges through the entire patient care process [23,24,25]. Furthermore, it shows potential to improve global healthcare inequalities by offering equal access to diagnostic, prognostic, and treatment approaches [26,27].

Incorporating AI tools into radiological and pathological workflows has been increasingly explored, indicating possible progress in neuro-oncology [28,29]. AI plays a crucial role in brain tumor analysis by providing a complete framework that incorporates machine learning (ML) and deep learning (DL) techniques, computer vision (CV), and their integration into computational biology. Machine learning methods in the field of artificial intelligence aid in the identification of patterns in imaging and genomic data. Deep learning, a specific branch of machine learning, particularly shines in extracting complex features. Using traditional image processing techniques and state-of-the-art deep learning approaches, computer vision accurately analyzes visual data for precise medical picture interpretation. Computational biology utilizes ML and DL to examine large biological datasets, assisting in comprehending the genetic and molecular characteristics of brain cancers. Combining these techniques improves the comprehensiveness and precision of brain tumor characterization, impacting diagnosing, predicting the outcome, and planning the treatment.

A noninvasive therapeutic approach application of the three forms called radiomic features (RFs), which involves the application of artificial intelligence in MRI, exists to address this issue. Several studies examine the radiomic features’ ability to evaluate prognosis in glioblastoma [30,31,32,33,34,35,36,37,38,39,40,41,42,43]. RFs are a nascent discipline in medicine that focuses on extracting quantitative features from radiographic images. These traits are invisible to the naked eye but possess the capacity to define the diversity within a tumor [44]. Additionally, in intratumoral heterogeneity, radiomics is often termed a “virtual biopsy” due to its capacity to enhance traditional diagnostic imaging by providing additional insights that are not visible to the naked eye and involve processes beyond the standard radiologic evaluation [45]. Nevertheless, traditional approaches that depend on the stage of the disease and clinical factors have several drawbacks, such as difficulties in interpretation, biases, and the requirement for large datasets. Conventional approaches still face difficulties in accurately predicting recurrence and survival for tailored care.

RF-AI is crucial in enhancing prognostic capacities in brain tumor care. Its approaches are increasingly used to forecast OS and PFS by utilizing characteristics derived from imaging data before therapy. Promising research has been conducted on the use of radiomic signatures derived from T1 and FLAIR MRI scans of glioblastoma patients, as well as T1, T2, and FLAIR scans of patients who have not received therapy [36,46,47]. These studies have shown substantial potential in predicting PFS and OS. The AI models surpass typical clinical characteristics and exhibit exceptional performance when paired with clinical parameters in glioblastoma patients [33,48]. Notably, models that utilize T2-weighted MRI and radiomic characteristics from peritumoral edema have shown connections with survival outcomes, site of recurrence, and molecular subtype, particularly in patients with glioma and glioblastoma. Deep learning models are developed to detect cancers and predict the location of recurrence, often before radiologists can detect it. These models, employing diverse imaging techniques, showcase RFs’ remarkable prediction prowess [40,49]. This study aims to systematically gather evidence and evaluate the prognosis significance of radiomics in glioblastoma using an RF-AI-based approach. In addition, a current study will be incorporated to complement the earlier research.

## 2. Materials and Methods

### 2.1. Literature Search

We extensively searched the PubMed, ScienceDirect, EMBASE, Web of Science, and Cochrane databases to identify relevant original research on applying RF-based AI in predicting glioblastoma outcomes. This search was finalized on 25 July 2024. Our search terms included glioblastoma, MRI, magnetic resonance imaging, radiomics, and survival or prognosis. We included only English-language studies involving human subjects. The retrieved references were organized using Mendeley Reference Manager v1.19.8, and the bibliographies of the chosen studies were further reviewed to uncover additional relevant articles. The characteristic of the study produced the subsequent findings: (1) the initial name and year of publication; (2) the nation and total sample; (3) the center of study; (4) treatment status; (5) mean age; (6) MR sequence protocol; (7) TCGA/TCIA dataset; and (8) feature extraction.

### 2.2. Data Selection

A study was undertaken to evaluate the application of radiomics, utilizing machine learning (ML) or deep learning (DL) techniques, for predicting the prognosis of glioblastoma patients. The inclusion criteria for selecting articles were as follows: (1) Patients must be diagnosed with glioblastoma. (2) The study must use a multiparametric brain MRI as the index test, with no prior treatment history, and include a detailed radiomic analysis. (3) OS and PFS were assessed using clinical and imaging follow-ups as the reference standard. (4) Both retrospective and prospective cohort studies were included. Due to the scarcity of recently published journals following the modifications, we cannot align our categorization with IDH1 according to the latest WHO 2021 recommendations [50]. While some still adhere to the old guidelines, we lack the necessary prognostic data to proceed with the analysis. The following criteria were used for exclusion: (1) case reports and case series; (2) papers that are reviews, editorials, letters, or abstracts; (3) studies that lack sufficient information on patient survival outcomes; (4) studies that do not include radiomic characteristics; (5) studies that conduct research with a group of patients who have some overlapping characteristics; (6) research investigations relying solely on publicly available data from sources like the Cancer Imaging Archive (TCIA) or the Cancer Genome Atlas (TCGA); (7) studies conducted without including independent internal or external validation, particularly cross-validation with the leaving-one-out method, and (8) studies utilizing C-index parameters. Two reviewers (D.P.W.W. and S.W.) independently assessed and selected the most appropriate studies using a standardized form. This study is registered with PROSPERO under CRD42024565289. The publication was subsequently developed following the PRISMA principles.

### 2.3. Definition of Variable

The patients’ prognoses were determined using either OS or PFS. OS is the time between the initial pathological diagnosis and death from any cause, while PFS is the time until the disease worsens. Patients still alive at the end of the follow-up period were considered right-censored.

The findings of this study included handcrafted radiomic features and deep learning models (DL). Machine learning (ML) integrated into handcrafted radiomic features is a growing trend in utilizing manually created features as input for machine learning models to enhance the accuracy and categorization of diseases. This integration uses the knowledge and skills involved in feature design and improves analytical capabilities through automated techniques. Deep learning models are developed to detect cancers and predict the location of recurrence, often before radiologists can detect it. These models, employing diverse imaging techniques, showcase the fantastic prediction prowess of AI. This deep learning system predicts partial transcriptional profiles and prognosis from histology images. This provides valuable insights into the potential of artificial intelligence in understanding intricate elements of tumor behavior.

### 2.4. Data Extraction

A generated Google Sheet in Excel Online retrieved the relevant data. To address any data gaps or inquiries, we initiated an electronic correspondence with the writers via email to collect the required information and two reviewers (D.P.W.W. and S.W.) assessed it independently.

### 2.5. Quality Assessment

The quality of the selected studies was assessed independently by two reviewers (D.P.W.W. and S.M.) utilizing the Newcastle–Ottawa Scale (NOS) [51] to determine the methodological rigor of the articles. The studies were classified into two quality categories: those rated between 4 and 6 were considered moderate-, while those rated between 7 and 9 were deemed high-quality. NOS is the bias quality of the study using the Cochrane risk of bias assessment methodology. Any reviewer disagreements were resolved through open discussion and consensus, with the involvement of a third author (S.W.).

### 2.6. Statistical Analysis

Hazard ratios (HRs) and their 95% confidence intervals (CIs) for overall survival (OS) and progression-free survival (PFS) were combined using random effects models. Publication bias was evaluated using the Egger test, with a *p*-value less than 0.05 suggesting the presence of bias. A forest plot was created to present the results of the statistical analysis. The random effects model is employed in statistical analysis to accurately reflect the underlying population’s calculation. This study used Review Manager 5.3 version software developed by RevMan Cochrane in London, UK.

## 3. Results

### 3.1. Literature Search

**Figure 1:**
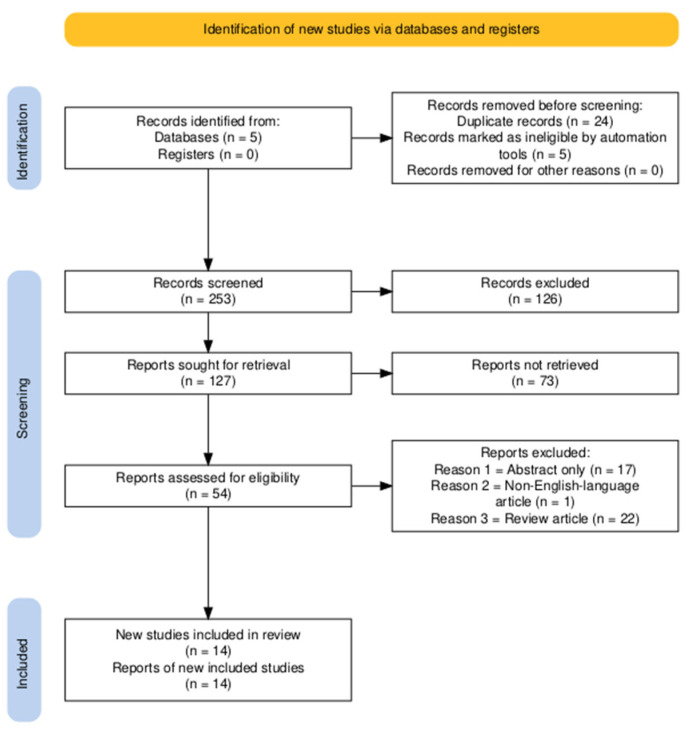
PRISMA diagram of the selection process.

Figure 1 displays a flow diagram illustrating the selection procedure for eligible studies. A total of 253 studies were found in the initial search across the three databases. Before screening, we eliminate 29 duplicate studies. We searched five databases, and of the 54 articles, 40 were excluded due to not meeting the eligibility criteria. The studies we discovered examined individuals with an average age ranging from 47 to 62 years. We found several publications that were unsuitable due to their use of a language other than English (*n* = 1), articles that only included abstracts (*n* = 17), and articles that were classified as reviews (*n* = 22). The fourteen included publications [30,31,32,33,34,35,36,37,38,39,40,42,43,52] consist of four from South Korea and China, two from Germany and the USA, and one from the UK and France, respectively. A total of 11 papers employed conventional treatment, with 5 being multi-center studies, while the remaining 9 utilized single-center research. The sample sizes of these studies varied from 22 to 652 people. A grand number of 2.950 samples took part in this study. The hazard ratios (HRs) and 95% CIs were directly obtained from the source articles in the investigations mentioned above. Table 1 presents the attributes and qualities of the studies that have been enumerated.

### 3.2. Quality Assessment and Bias Analysis

According to the NOS quality evaluation, the investigations shown in Table 1 were determined to have moderate to high quality. Egger’s tests revealed that publication bias did not influence the included studies. The investigations revealed a p-Egger with an OS rate of 1.536 and a PFS rate of 1.994.

### 3.3. Overall Survival (OS) Analysis

**Figure 2:**
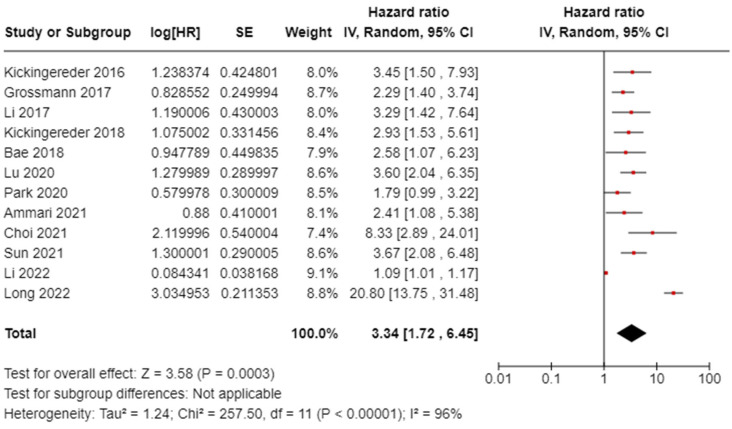
Overall survival (OS) analysis of the included studies [30,31,32,33,34,35,36,39,40,41,42,43].

We examined the analysis of OS in Figure 2 in glioblastoma. All twelve OS studies were considered, involving 1.639 patients from Germany, the USA, China, South Korea, the UK, and France. The random effects model was used to calculate the pooled HR for OS. The HR for OS was 3.34 (95% confidence interval [CI], 1.72–6.45). The analysis revealed heterogeneity in the OS with an *I*^2^ value of 96%.

### 3.4. Progression-Free Survival (PFS) Analysis

**Figure 3:**
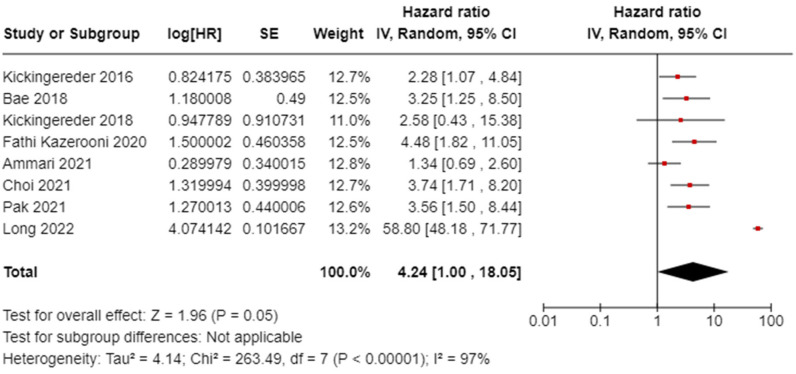
Progression-free survival (PFS) analysis of the included studies [30,33,34,36,37,38,39,42].

We examined the analysis of PFS in Figure 3 in glioblastoma. All eight PFS studies were considered, involving 747 patients from Germany, South Korea, China, the USA, and France. The random effects model was used to calculate the pooled HR for PFS. The HR for PFS was 4.24 (95% confidence interval CI, 1.00–18.05). The analysis revealed heterogeneity in the PFS with an *I*^2^ value of 97%.

## 4. Discussion

The health industry is witnessing technological advancements leading to the emergence of tools like AI, particularly in neurosurgery. This study indicated that patients exhibiting high-risk radiomic characteristics have worse prognoses, with pooled hazard ratios of 3.59 and 4.20 for overall survival and progression-free survival, respectively. AI is utilized in applying tools like RFs. Several types of artificial intelligence, such as machine learning and deep learning [28,53], have been developed from some RFs. Implementation in this domain significantly enhances information processing speed and improves the patient’s treatment regimen and overall quality of life. None of the publications we encountered provided information regarding the specific ML and DL techniques employed. Nevertheless, various categories of machine learning exist, including Support Vector Machines (SVMs) and DL-like Convolutional Neural Networks (CNNs), which are frequently used. The field has seen significant evolution due to the advancement of deep learning and other machine learning techniques. To summarize, the application of artificial intelligence in medical imaging involves using the sophisticated algorithms above to carry out tasks such as tumor detection and segmentation. These techniques improve the precision and speed of diagnosing medical conditions by automating and enhancing image analysis processing.

RF is anticipated to play a significant role in precision medicine due to its capability to collect detailed data that precisely characterize survival in glioblastoma, a notably aggressive cancer. Regardless of success or failure, the treatment outcome holds substantial importance, although other factors may also affect long-term survival. Combining Ktrans and relative cerebral blood volume metrics from perfusion-weighted imaging (PWI) sequences achieved an accuracy of approximately 91% [54]. In addition, the diagnostic accuracies of these algorithms exceeded 70%, outperforming neuroradiologists in evaluating typical MR images using algorithms [52,55]. A strong association was seen in patients with low-grade gliomas between the T2-weighted RFs of PFS and its functions, such as cell proliferation, apoptosis, immune response, and vascular development [56].

Despite its robust predictive capacity, integrating this therapeutic tool into practice has yet to be accomplished. The current application is restricted to conventional MRI, which is typically appraised by radiologists. RFs have computational limits and can be time-consuming, particularly during preprocessing [57]. This might be problematic when patients require prompt action or treatment. We removed certain studies that utilize datasets obtained from TCIA/TCGA to prevent the inclusion of redundant data, although they may have a distinct therapeutic approach. Consequently, the process of choosing articles was conducted with excellent adherence. The necessity for the standardization and calibration of imaging regulation was acknowledged, particularly in cases where the RFs can be replicated, and has recently been validated in multi-center experiments [58]. However, the study we analyzed needed to disclose whether it had undergone standardization with the latest standards despite being conducted after the research.

This systematic review and meta-analysis thoroughly assessed the predictive significance of RFs in individuals diagnosed with glioblastoma. Our study showed that individuals with radiomic signs indicating a high risk have worse prognoses, with HRs of 3.59 and 4.2 for OS and PFS, respectively, compared to patients without these features. None of the studies exhibited publication bias. The results of our study indicate that the use of RFs can accurately assess the risk level of patients with glioblastoma at an early stage. This information can help physicians develop more effective treatment plans and potentially enhance patients’ chances of recovery. We chose not to utilize the C-index parameter because it frequently requires comparing patients with similar underlying risks, which can result in numerous comparisons. Additionally, the pairwise comparisons of patients with possibly similar risks yield almost equal results. The factors above contribute to the findings corroborated by prior research. Specifically, the analysis using the C-index reveals heterogeneity that differs among investigations [59].

Glioblastoma imaging currently requires frequent imaging sessions. The current status of radiomic profiling is most effective during the initial scans following diagnosis, as it can potentially guide decisions regarding radiotherapy or surgical resection [60]. The clinical significance of these profiles will be enhanced if they can be associated with an identifiable histological pattern. Each study can characterize a distinct phenotype or microenvironment. Additional histological validation with a larger sample size is required. While we saw publications discussing edema, recurrence, and metastasis, they did not fit our inclusion criteria. However, treatment effects can alter the volume, threshold, or profiles that are most valuable in predicting prognosis. By monitoring the expansion of the profile over time, clinicians can effectively manage and account for both the impacts of treatment and the influence of time. Temporal fluctuations in prognostic scores can offer valuable therapeutic insights.

Nevertheless, this study’s limitation is that radiomics is constrained by the absence of standards, impeding the ability to reproduce results. An inherent limitation of this study is that most studies employ retrospective designs, yet this study shows promise as a future prediction tool. Consequently, it is essential to create standard methods for both imaging acquisition and segmentation. In conclusion, using RFs shows excellent potential in accurately describing glioblastoma-based artificial intelligence. This is especially true when combining multiple modalities and considering the clinical presentation of the patients. We all expect that RFs can play a crucial role in long-term forecasting and planning for survivorship care, assisting in making treatment decisions. Facilitating the connection between clinical practice and research via data-sharing networks can expedite the creation and validation of AI models. The transparency and interpretability of AI models are crucial for establishing confidence and acceptance in therapeutic environments.

## 5. Conclusions

An RF-AI-based approach offers prognostic significance for OS and PFS in patients with glioblastoma. Acquiring more detailed data on patient demographics, treatment responses, and illness features can augment AI model performance. Additionally, designing research with higher sample sizes and different patient populations can also boost precision and application. From an economic standpoint, this type of AI has the potential to significantly benefit developing countries where access to healthcare is costly and where individuals may lack the financial resources for MGMT checks.

## Figures and Tables

**Table 1:** Baseline study characteristics.

Study	Nation	Total No. of Patients	Center of Study	Treatment Status	Mean Age ± SD (Range)	MR Sequence Protocol	TCGA/TCIA Dataset	Feature Extraction	NOS
Kickingereder 2016 [36]	Germany	119	Single-center	Standard treatment	NA	T1, FLAIR	No	Handcrafted radiomic features	8
Grossmann 2017 [35]	USA	291	Multi-center	Standard treatment	57 (24–88)	T1CE, FLAIR	No	Handcrafted radiomic features	9
Li 2017 [43]	China	92	Multi-center	NA	55 (10–81)	T1, T2, FLAIR, T1CE	Yes	Handcrafted radiomic features	8
Bae 2018 [34]	South Korea	217	Single-center	Standard treatment	58 ± 12	T1CE, FLAIR, T2, ADC	No	Handcrafted radiomic features	8
Kickingereder 2018 [33]	Germany	181	Single-center	Standard treatment	62 (54–71)	T1, T2, FLAIR	No	Handcrafted radiomic features	7
Fathi Kazerooni 2020 [38]	USA	80	Multi-center	Standard treatment	57 (22–86)	T1, T1CE, T2, FLAIR, DTI, DSC	No	Handcrafted radiomic features	8
Lu 2020 [32]	UK	181	Single-center	Standard treatment	60 ± 13	T1CE	No	Handcrafted radiomic features	8
Park 2020 [31]	South Korea	216	Multi-center	Standard treatment	58 (20–80)	T1CE, FLAIR, DWI, DSC	No	Handcrafted radiomic features	8
Ammari 2021 [30]	France	194	Single-center	Standard treatment	57 (18–80)	T1CE, FLAIR	No	Handcrafted radiomic features	8
Choi 2021 [42]	South Korea	120	Single-center	Standard treatment	57 ± 14	T1CE, FLAIR, T2	No	Handcrafted radiomic features	7
Pak 2021 [37]	South Korea	150	Single-center	Standard treatment	61 ± 14	DCE	No	Handcrafted radiomic features	8
Sun 2021 [41]	China	435	Multi-center	Standard treatment	55 ± 15	T1, T2, FLAIR, T1CE	Yes	Handcrafted radiomic features	9
Li 2022 [40]	China	652	Single-center	Pre-operative	NA	T2	Yes	Deep learning	8
Long 2022 [39]	China	22	Single-center	Pre-operative	47 (23-67)	T1, FLAIR	Yes	Handcrafted radiomic features	8

Legend: NOS = Newcastle–Ottawa Scale; NA = Not available.

## Data Availability

This article encompasses all data produced or analyzed during the study. For additional inquiries, please contact the corresponding author.
